# Effects of Extreme Climate Events on Tea (*Camellia sinensis*) Functional Quality Validate Indigenous Farmer Knowledge and Sensory Preferences in Tropical China

**DOI:** 10.1371/journal.pone.0109126

**Published:** 2014-10-06

**Authors:** Selena Ahmed, John Richard Stepp, Colin Orians, Timothy Griffin, Corene Matyas, Albert Robbat, Sean Cash, Dayuan Xue, Chunlin Long, Uchenna Unachukwu, Sarabeth Buckley, David Small, Edward Kennelly

**Affiliations:** 1 Sustainable Food and Bioenergy Systems Program, Department of Health and Human Development, Montana State University, Bozeman, Montana, United States of America; 2 Department of Biology, Tufts University, Medford, Massachusetts, United States of America; 3 College of Life and Environmental Sciences, Minzu University of China, Beijing, China; 4 Department of Anthropology, University of Gainesville, Gainesville, Florida, United States of America; 5 Friedman School of Nutrition Science and Policy, Tufts University, Boston, Massachusetts, United States of America; 6 Department of Geography, University of Gainesville, Gainesville, Florida, United States of America; 7 Department of Chemistry, Tufts University, Medford, Massachusetts, United States of America; 8 Department of Biochemistry, The Graduate Center of the City University of New York, New York, New York, United States of America; 9 School of Engineering, Tufts University, Medford, Massachusetts, United States of America; CSIRO, Australia

## Abstract

Climate change is impacting agro-ecosystems, crops, and farmer livelihoods in communities worldwide. While it is well understood that more frequent and intense climate events in many areas are resulting in a decline in crop yields, the impact on crop quality is less acknowledged, yet it is critical for food systems that benefit both farmers and consumers through high-quality products. This study examines tea (*Camellia sinensis*; Theaceae), the world's most widely consumed beverage after water, as a study system to measure effects of seasonal precipitation variability on crop functional quality and associated farmer knowledge, preferences, and livelihoods. Sampling was conducted in a major tea producing area of China during an extreme drought through the onset of the East Asian Monsoon in order to capture effects of extreme climate events that are likely to become more frequent with climate change. Compared to the spring drought, tea growth during the monsoon period was up to 50% higher. Concurrently, concentrations of catechin and methylxanthine secondary metabolites, major compounds that determine tea functional quality, were up to 50% lower during the monsoon while total phenolic concentrations and antioxidant activity increased. The inverse relationship between tea growth and concentrations of individual secondary metabolites suggests a dilution effect of precipitation on tea quality. The decrease in concentrations of tea secondary metabolites was accompanied by reduced farmer preference on the basis of sensory characteristics as well as a decline of up to 50% in household income from tea sales. Farmer surveys indicate a high degree of agreement regarding climate patterns and the effects of precipitation on tea yields and quality. Extrapolating findings from this seasonal study to long-term climate scenario projections suggests that farmers and consumers face variable implications with forecasted precipitation scenarios and calls for research on management practices to facilitate climate adaptation for sustainable crop production.

## Introduction

Climate change is impacting agro-ecosystems, livelihoods, and human wellbeing in communities worldwide. In some cases, crop production is at risk due to droughts, wildfires, floods, severe temperature fluctuations, and other extreme weather conditions reducing yields and shifting the geographic range where crops can be cultivated [1], [2]. In contrast, increasing temperatures in the higher latitudes in both the northern and southern hemisphere are enhancing the yield of some crops [3]. While the impact of climate change on crop yield has been well documented [1], [2], [3], [4], [5], it is also important to understand potential changes in crop quality on farmer livelihoods and food security [6]. This is particularly important for many specialty crops such as fruits, vegetables, herbs, coffee, chocolate, and tea where functional quality is determined by phytonutrients and secondary metabolites, or bioactive food components, that benefit consumers. Bioactive food components can potentially enhance flavor profiles and other sensory experiences [7] and also have the potential to mitigate micronutrient deficiencies and associated risks of chronic disease [6], [8]. In addition, producer income can benefit from increased consumer preference for high-quality crops generated through premiums reflected in higher prices. This study uses tea (*Camellia sinensis*; Theaceae), the world's most widely consumed beverage after water, as a case study to explore effects of extreme climate events on crop quality and associated farmer knowledge, preferences, and income.

China is the world's largest supplier of tea, where its production is vulnerable with many of the country's tea agro-ecosystems located in high-risk regions for climate change [9]. In addition, China has an arable land shortage with land suitable for cultivation comprising only 7% of the world's total to support 22% of the global population [10]. Farmer livelihoods in China are at risk with shifts in climate conditions that support their crops. Precipitation and temperature changes have been recognized to impact the yield and geographic range of many crops including tea plants [11], [12]. Climate change may also influence tea functional quality that is largely determined by the concentrations of the methylxanthine caffeine and various polyphenolic catechin compounds that are respectively responsible for tea's stimulant, antioxidant, anti-inflammatory, and cardioprotective properties [13].

The secondary metabolites that contribute to crop quality serve as defense compounds in plants and vary in concentration depending on genetic, environmental, and management conditions. For example, the concentrations of methylxanthine and polyphenolic catechin compounds in tea plants vary with geographic location, cultivar, herbivory, season, shade, soil, slope, water availability, and management [13], [14], [15]. Consumers can perceive changes in the concentrations of tea functional compounds by their sensory characteristics, including astringency, bitterness, and sweetness [7]. Ultimately, crop quality influences consumer-purchasing decisions, farmer livelihoods, and functional benefits derived from crops.

The impacts of climate change are likely to have a notable impact on tea farmers with tea production supporting the livelihoods of millions of farmers and their communities - a growing global market of over $20 billion USD annually. Farmers can mitigate climate effects on crop quality through adaptive management. However, in order for adaptation to occur, farmers must first have knowledge of *how* the climate has changed [16] and impacted their crops. Previous research evaluating individual perceptions of climate change based on seasonal patterns has proven useful for the study of climate change [17].

The present paper integrates plant-defense and socio-ecological theoretical foundations through a coupled natural and human systems framework to examine impacts of extreme climate events on crop quality and socio-economic responses using tea production in China as a study system. Field studies during extreme climate events such as droughts and flooding offer the opportunity to examine effects of climate variability in natural settings under unusual circumstances that are expected to become more frequent and severe with forecasted climate change. Previous work that highlights the underestimation of manipulative studies on plant responses to climate change compared to observational seasonal studies points to the need for research in natural settings [18]. For the present study, we utilized one of the most severe droughts that occurred in recorded history at our study site in China's Yunnan Province during the spring tea harvest along with the onset of the East Asian monsoon that marks the start of the monsoon tea harvest as opportunities to examine the effects of extreme climate events that are expected to become more prevalent in tea producing areas.

**Figure 1:**
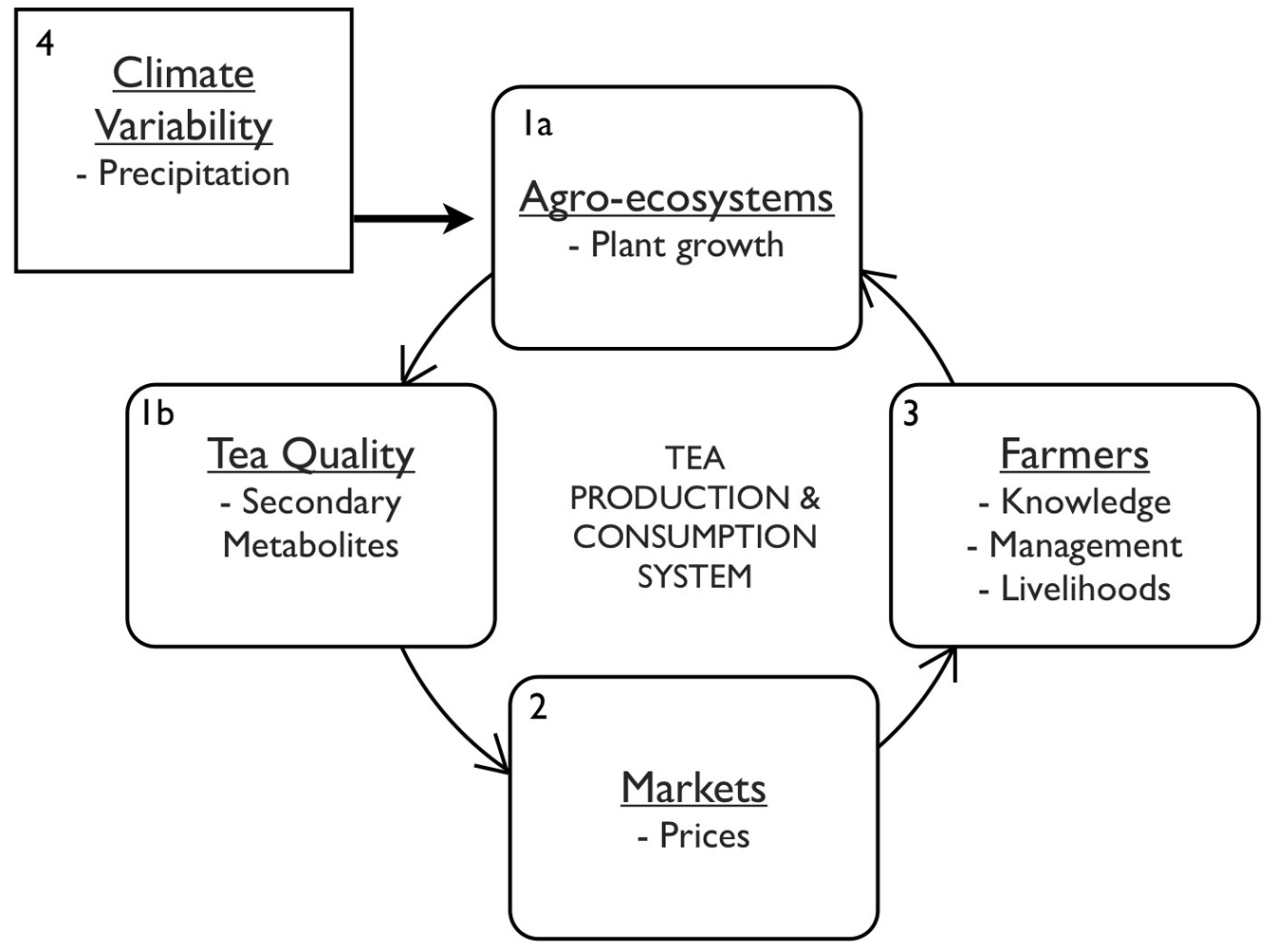
Tea Production and Consumption System. Our study objectives are to examine the tea production and consumption system on the basis of the following dimensions: (1) effects of precipitation variability between an extreme drought that occurred during the dry spring tea harvest season and monsoon tea harvest season on growth and functional quality of tea leaves, (2) tea prices during the dry spring tea harvest season and monsoon tea harvest season, (3) farmer knowledge of climate effects on tea quality and associated management, sensory preferences, and livelihoods and, (4) precipitation trends over the past 40 years as well as forecasted precipitation at the study site.

Specifically, our study objectives are to provide quantitative data on how climate effects the following aspects of the coupled tea production and consumption system (Figure 1): (1) the impact of seasonal precipitation variability that occurs between the spring drought and monsoon on tea growth and functional quality, (2) variation of tea prices between the spring drought and monsoon tea harvests, (3) farmer knowledge of climate effects on tea quality and associated management, sensory preferences, and livelihoods and, (4) precipitation trends over the past 40 years as well as future climate scenario projections at the study site. We hypothesize the following: (1) the increase in precipitation that occurs with the seasonal transition from the spring drought to the monsoon tea harvests results in an increase in tea yields and a decrease in functional quality, (2) tea prices decrease with a drop in quality resulting from increased precipitation between the spring drought and monsoon tea harvests, (3) farmers at the study site perceive climate variation, have knowledge of climate effects on tea quality, and have sensory preferences on the basis of personal experiences and interactions with the tea market and, (4) precipitation patterns at the study site have varied over the past 40 years and are expected to continue to vary with drought and heavy monsoon rains becoming more extreme and frequent with climate scenario projections.

## Materials and Methods

### Ethics Statement

Household surveys were conducted according to a human subjects protocol approved by the ethics committee at Minzu University of China. Participants provided their written informed consent to participate in this study. No unique identifiers were attached to the survey data. Permission to conduct interviews and sample tea leaves at the study site was granted by the Secretariat of the Communist Party in Menghai County, Xishuangbanna Dai Autonomous Prefecture, Yunnan Province, China. Sampling of tea leaves was carried out on a private commercialized tea plantation with the permission of the smallholders managing the plantation. No sampling was conducted in a protected area and no wildlife was sampled for this study.

### Study site

This project was carried out in an indigenous Akha small-landholder farming community located in evergreen broadleaf montane forest at an altitude between 1 600–1 850 m in Menghai County, Xishuangbanna Dai Autonomous Prefecture in Yunnan Province of southwest China. The study area is situated on the northern edge of the tropical zone in Southeast Asia [19] within the Indo-Burma biodiversity hotspot. The Akha are a Tibeto-Burmese socio-linguistic group that migrated to the region several hundred years ago from the Tibetan plateau. The population at the study site is approximately 400 inhabitants living in family units of a total of 100 households. Each household manages 10 to 30 land-use types including tea agro-forests and monoculture terraces [20]. The study community has been cultivating tea for hundreds of years and produces a premium product sought out by urban connoisseurs in China and elsewhere. Income at the study site is primarily derived from the cultivation, processing, and sale of premium tea to traders and vendors. The study region is characterized by an annual mean air temperature of approximately 22°C, annual precipitation between 1 400–1 500 mm and, a pronounced dry season of six months [19].

### Tea harvest seasons

Tea is a perennial plant that is harvested during three distinct seasons in the study area. The three harvest seasons at the study site are dependent on the onset and retreat of the East Asian Monsoon including: (1) a dry spring tea harvest (around mid-March until end of April to mid-May), (2) a monsoon tea harvest marked by the onset of the East Asian Monsoon when warm-wet air masses arrive from the Indian Ocean (around mid-May until end of September) and, (3) a dry autumn tea harvest marked by the retreat of the East Asian Monsoon and arrival of Continental air masses from subtropical origins (around October to mid-November).

### Tea growth and functional quality

Stratified time-series sampling was conducted by harvesting tea plants on a daily basis during three sub-seasonal harvest periods starting from the dry spring tea harvest to the monsoon tea harvest. The three harvests are dependent on precipitation with no significant differences in temperature. Daily precipitation and temperature was gathered at the study site during the study period using a weather station to delineate tea harvest seasons that are dependent on precipitation. Each sampling period included five days of sampling corresponding to a tea harvest season or sub-season as follows: (1) spring drought tea harvest from 8–12 May 2012, (2) monsoon onset tea harvest from 13–18 May 2012 and, (3) monsoon tea harvest from 20–25 May 2012.

Sampling was carried out in terrace monoculture teagardens at the study site managed by local smallholders. Three 15 m×15 m plots each consisting of 100–120 tea plants were marked in terraced monoculture teagardens on the same mountain slope at altitudes ranging from 1 787–1 810 m. Tea plants were randomly labeled within each plot. Five tea plants per plot were harvested on each sampling day. No tea plants were harvested more than once to maintain sample independence and to limit the possibility of induction of secondary metabolite defense compounds by leaf removal. For each tea plant, we collected twenty harvest units each consisting of one bud and the adjacent two leaves from various positions on tea plants using sharp shearing scissors. The length of the first fully expanded leaf was measured on ten of the twenty leaves of each sample. All 20 harvest unit samples from an individual plant were combined for secondary metabolite analyses. Leaf samples were processed within two hours of collection to deactivate oxidative enzymes and dried in a microwave oven (700 watts; Haier Electronics Group, Hong Kong) on the basis of an adapted green tea processing protocol [21]. Processed tea leaves were stored in sealed plastic bags in dark conditions at room temperature in the field. Samples were brought to the laboratory, weighed, and sequentially frozen after 20 days from harvest so that each sample was stored at room temperature for the same time period.

Samples were ground to a fine powder in a ball mill and twenty mg of dried leaves were extracted in 1.5 mL 80% aqueous HPLC-grade methanol (Fisher Scientific, Fair Lawn, NJ). The resulting mixture was vortexed for 30 sec (Genie 2, Fisher Scientific, Fair Lawn, NJ) and sonicated for 30 min at 20°C (Quantrex 280, L&R Ultrasonics) and then centrifuged for 15 min at 15 000 rpm (Marathin Micro A, Fisher Scientific).

We measured the concentration of eight antioxidant polyphenol compounds and three methylxanthine compounds that are linked to tea functional quality including its sensory characteristics, health claims, and stimulant properties. The analytes measured for tea functional quality in this study are those recognized by the International Organization for Standardization as well as by the FAO Intergovernmental Panel on Tea's Standards for Tea. Target antioxidant polyphenols included catechin (C), catechin gallate (CG), epicatechin 3-gallate (ECG), epigallocatechin (EGC), epigallocatechin 3-gallate (EGCG), gallic acid (GA), gallo catechin (GC), and gallocatechin 3-gallate (GCG). Target methylxanthines included caffeine (CAF), theobromine (TB) and theophylline (TP). Total methylxanthine content (TMC) was reported from HPLC data by the addition of individual amounts of methylxanthine compounds. All standards were purchased from ChromaDex (Santa Ana, CA).

High Performance Liquid Chromatography (HPLC) was performed as described by some of the study's authors in a previous paper [22]. The method was validated with respect to accuracy, precision, sensitivity, and selectivity. A Waters 2695 (Milford, MA) module HPLC equipped with a 996 photodiode array detector and a 4 µm, 250×4.6 mm ID, C-18 Synergi Fusion, reversed-phase column (Phenomenex, Torrance, CA) was used. Column and autosampler temperatures were maintained at 38°C and 4°C, respectively. Five µL were injected for each sample. The mobile phase was 0.05% (v/v) trifluoroacetic acid in distilled water (Solvent A) and 0.05% (v/v) trifluoroacetic acid in acetonitrile (Solvent B). The following gradient was used at a flow rate of 1 mL/min: 12–21% Solvent B from 0–25 min; 21–25% Solvent B from 25–30 min. The column was flushed with 100% Solvent B for 10 min at the end of each run and was re-equilibrated for 5 min to starting conditions. Spectra were recorded from 254 to 400 nm and relevant peaks were detected at 280 nm on the basis of characteristic absorbance spectra and retention time. Analyte concentrations were determined using peak areas and the linearity determined by plotting signal versus concentration standard curve equations with the limit of detection (LOD) and the limit of quantification (LOQ) in the ranges of 0.05–1 and 0.1–5 g ml^−1^, respectively.

Total phenolic concentration (TPC) was determined spectrophotometrically using Folin–Ciocalteau reagent as described in a previous paper [22]. Samples were analyzed in triplicates. Absorbance values were measured at 765 nm using a Benchmark Plus Microplate Spectrometer (Bio-Rad, Hercules, CA) and results expressed as gallic acid equivalents (GAE) in mg g^−1^ dry plant material. The concentration of polyphenols in tea samples was derived from a standard curve of gallic acid concentration vs. absorbance between 31.25–500 g ml^−1^.

Anti-oxidant activity of tea extracts was assessed with the 1-1-diphenyl-2-picrylhydrazyl (DPPH) free radical scavenging capacity assay as described in a previous paper [22]. Gallic acid (0.03–0.25 mg ml^−1^) and ascorbic acid (0.03–0.50 mg ml^−1^) were used as positive controls. Each mixture was prepared in quadruplicates of five concentrations. Absorbance values were measured at 517 nm using a Benchmark Plus Microplate Spectrometer (Bio-Rad, Hercules, CA). Radical scavenging capacity of each sample was calculated as the percentage of DPPH free radicals inhibited by the sample in comparison to radical inhibition in the negative water control. Values obtained were plotted against concentration (µg ml^−1^). Free radical scavenging capacity was expressed as IC_50_ values (concentration of sample required to scavenge 50% of DPPH radicals; the lower the IC_50_ value, the higher the anti-oxidant activity).

### Farmer knowledge, sensory preferences, and tea prices

Participant observation and open-ended surveys were carried out in order to develop a structured survey instrument. Structured household interviews using the developed survey instrument were conducted to assess cultural consensus of farmer perceptions, experiences, and management adaptations associated with impacts of climate change and extreme weather events on tea agro-ecosystems and quality. Additional structured household interviews were conducted with smallholder tea farmers at the study site to determine tea prices associated with the spring tea harvest and the monsoon tea harvest over the past decade.

Cultural consensus analysis is a mathematical model that uses factor analysis to test informant agreement regarding a cultural domain on the basis of informant responses to systematic interview questions [23]. It is based on the finding that agreement indicates knowledge within a particular cultural domain. Within the model, consensus of knowledge is met if the ratio of the eigenvalues for the first and second factor is >3∶1. The utility of the method lies in its ability to quantify variation, illuminate instances of disagreement, and find the culturally “correct” answer to a particular question. Cultural consensus analysis also allows for a relatively small sample size to provide a statistically high level of confidence in responses. For example, 29 informants with a 0.5 average level of cultural competence provide a 0.99 confidence level [24].

We developed interview questionnaires from pilot testing of sample questions at the study site and surrounding communities. The survey instrument consists of 23 questions that elicit structured responses that are each followed by prompts for open-ended responses. Survey questions are organized into four sections on perceptions and experiences related to: (1) season, precipitation, and temperature, (2) impacts of precipitation and temperature on crop quality, yields, pests, and weeds in tea agro-ecosystems, (3) impacts of climate on income and, (4) management adaptations to climate variability. Surveys were carried out with the assistance of translators trained in household survey methods.

We surveyed 32% of the 100 households at the study site on the basis of suitable sample sizes for cultural consensus analysis as reported in the literature [24]. One adult informant from each household was interviewed. We employed a stratified random design to ensure equal gender and age classes of informants. Age classes are defined here as young to middle-aged informants, whose age is less than 55 years old, and elder informants whose age is 55 years and older.

Data from the structured component of household surveys were analyzed in a binary matrix to determine cultural consensus between households in the community using UCInet 6 [25]. Responses from the open-ended component of the survey were coded individually by two analysts and agreed upon to determine thematic prevalence of responses within the community.

### Precipitation variability: historical trends and projected scenarios

Precipitation variability at the study site was examined at three scales: (1) short-term seasonal variability between the spring drought through the onset of the East Asian Monsoon during the sampling period of this study, (2) historical trends over a thirty-year range and, (3) precipitation scenario projections in the 2050-2079 period. Historical trends were calculated using long-term precipitation satellite data for the study site from the pentad (5-day) mean CMAP product over a thirty-year range [26]. The pentad mean precipitation rate is an estimate compiled from satellite, rain gauges, and the output of numerical weather prediction on a 2.5° grid. Satellite data was used because the nearest rain gauge is over 500 km from the study site and is less than 60 percent complete. The precipitation time series used to delineate the wet season is an average of the pentad mean precipitation grids in the 5° by 5° region surrounding the study site. Here, we define the wet season as the period in which 90% of the annual precipitation occurs. The wet is assumed to begin on the pentad when the cumulative precipitation exceeds five percent of the annual total in each year and ends when it exceeds 95%. To validate the models used, we plotted the CMAP data and the modeled data to calculate a measure of temporal overlap of different threshold values.

The significant regional differences in climate change scenario projections produced by different climate models make it difficult to interpret results of individual experiments. Instead of using small ensembles of a single model, we present maps of simulated changes in precipitation for the 21^st^ Century, estimated from an ensemble of single runs from 19 models used in the IPCC AR4 report [27]. The ensemble is the mean of run number one from all of the models. We estimated the mean precipitation rate from the ensemble mean of the 20^th^ Century runs and compared the mean precipitation rain rate of the dry spring season and the monsoon season over the following five-year periods: (1) 1979–1984, (2) 1985–1989, (3) 1990–1994, (4) 1995–1999, (5) 2000–2004 and, (6) 2005–2010. We then employed the ensemble mean of the SRES A1B scenario runs to create future climate scenarios for 2050–2079 to understand possible climate change scenarios and their implications for tea quality and farmer livelihoods at the study site.

### Statistical analysis

A Fit Model using a Standard Least Square Means personality function and Analysis of Variance was performed using JMP 10.0 (SAS Institute Inc.) to determine how leaf growth and tea functional quality parameters vary with increasing precipitation from the spring drought through the monsoon onset and monsoon tea harvests. Least Square Means of tea growth values, functional quality values, and farmer perceptions of tea quality (categorized as high, low or medium quality) were plotted against the three tea harvest sampling periods. A multiple comparison using the Least Square Means Tukey HSD method was applied to examine if tea growth and quality significantly increased or decreased with increasing precipitation from the spring drought through the monsoon onset and monsoon tea harvests. Plot and sampling day were used as random variables. Finally, we applied regression analysis to evaluate the relationship between leaf growth measurements and secondary metabolite chemistry with plot as the unit of replication.

## Results

### Tea growth and functional quality

**Figure 2:**
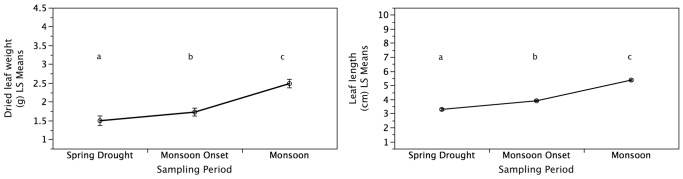
Effects of Precipitation Variability on Tea Growth. Increased precipitation from the spring drought to the monsoon tea harvest significantly increased tea leaf weight and length. Sampling periods not connected by the same letters are significantly different. Values are means ± one standard error.

Tea leaves harvested during the spring drought appeared drier compared to the leaves sampled during the monsoon onset and monsoon tea harvests that appeared tender. Leaf length (n = 2,250) and leaf weight (n = 225) increased significantly (*P*<0.01) with the increase in precipitation over the sampling periods from the spring drought to the monsoon onset and monsoon onset to monsoon tea harvests (Figure 2).

**Figure 3:**
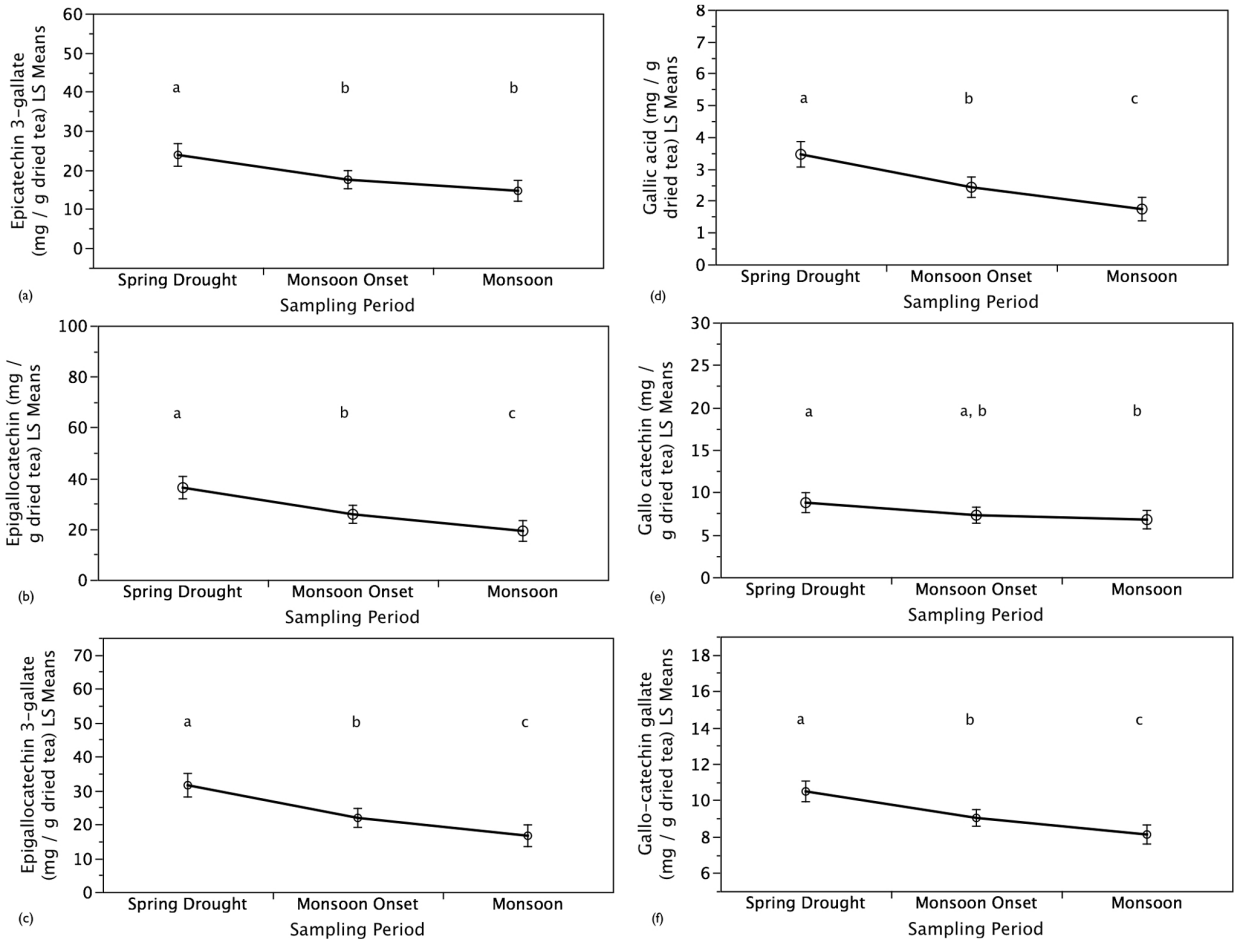
Effects of Precipitation Variability on Tea Polyphenolic Catechins. Increased precipitation from the spring drought to the monsoon tea harvest resulted in significantly lower concentrations of (a) epicatechin 3-gallate (ECG), (b) epigallocatechin 3-gallate (EGCG), (c) epigallocatechin (EGC), (d) gallic acid (GA), (e) gallocatechin (GC), (f) gallocatechin gallate (GCG) as well as catechin (C) and catechin gallate (CG; not shown). Sampling periods not connected by the same letters are significantly different. Values are means ± one standard error.

**Figure 4:**
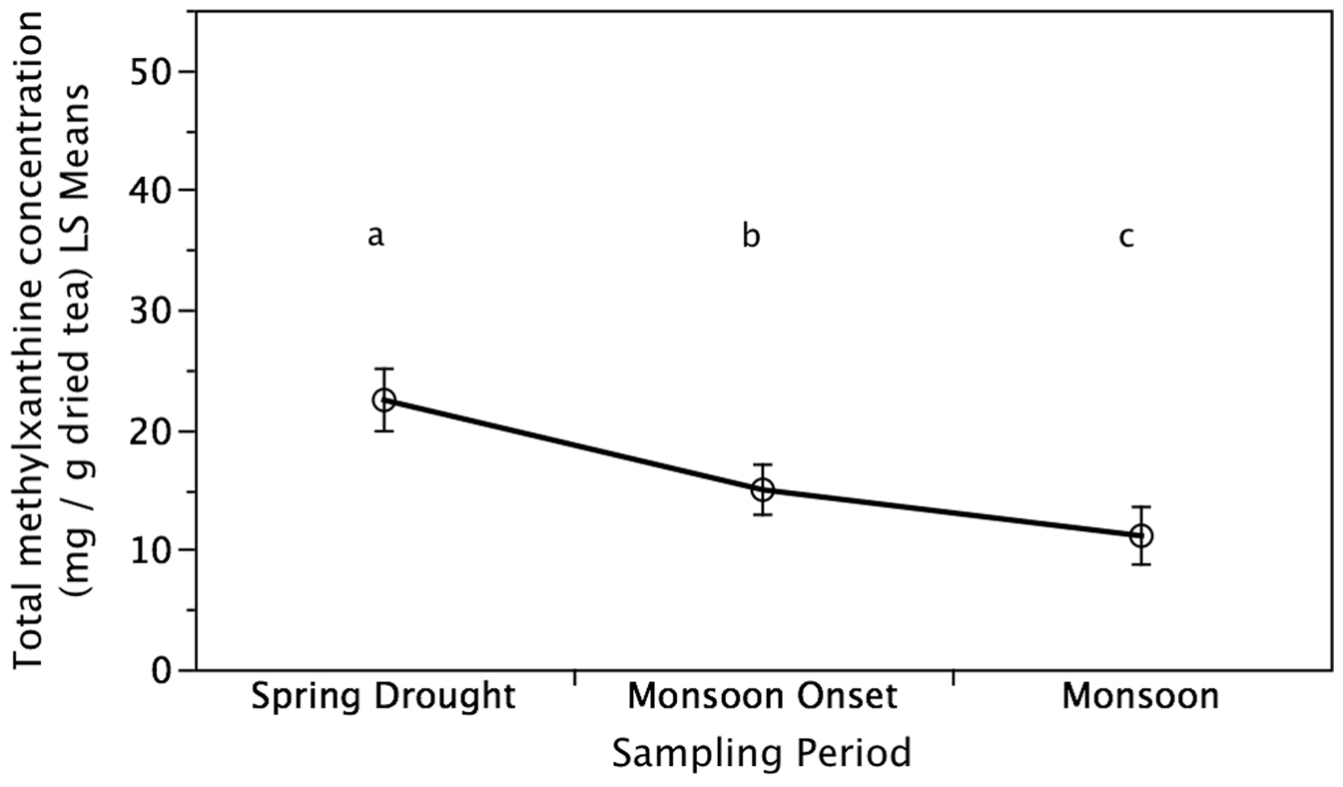
Effects of Precipitation Variability on Total Methylxanthine Concentration. Increased precipitation from the spring drought to the monsoon tea harvest resulted in significantly lower total methylxanthine concentration (TMC) of tea leaves. Sampling periods not connected by the same letters are significantly different. Values are means ± one standard error.

**Figure 5:**
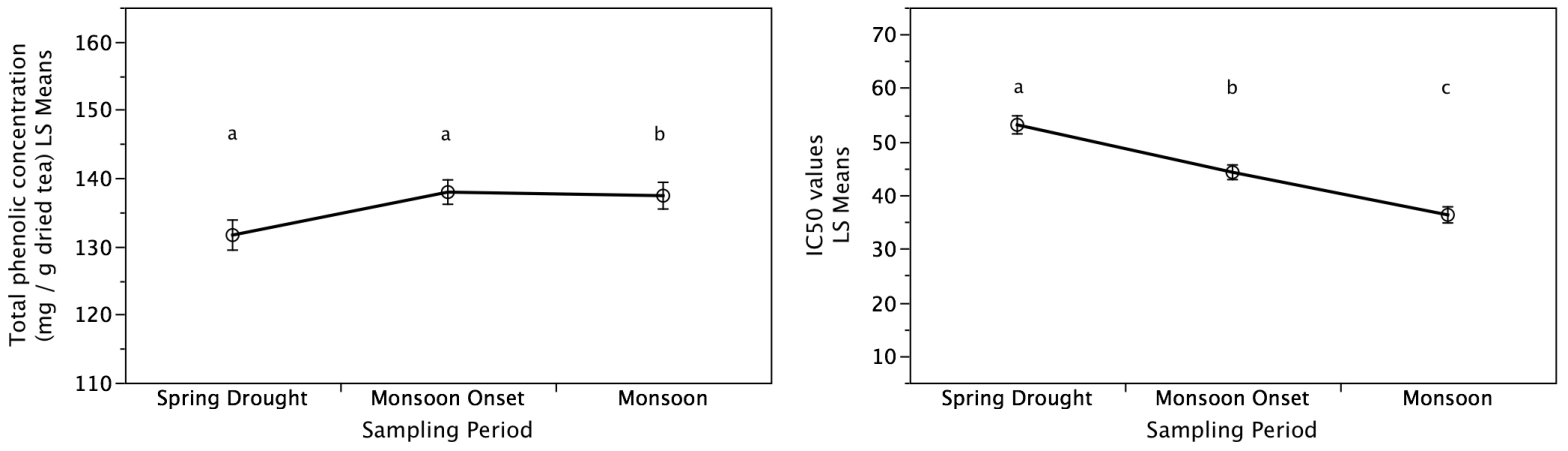
Effects of Precipitation Variability on Total Phenolic Concentration and Antioxidant Activity. Increased precipitation from the spring drought to the monsoon tea harvest resulted in significantly higher total phenolic concentration (TMC) and antioxidant activity of tea leaves. Sampling periods not connected by the same letters are significantly different. Values are means ± one standard error.

The concentration of six major tea polyphenolic metabolites (EGC, EGCG, ECG, GCG, C and GA) that determime tea functional quality decreased (*P*<0.05) with the onset of the East Asian Monsoon from the spring drought to the monsoon tea harvest (Figures 3). The compounds with the largest change in concentration during the transition from the spring drought to the monsoon tea harvest were EGC, EGCG, and ECG. These metabolites were also found to have the highest concentrations during all three seasonal tea harvest periods. In addition to changes in these polyphenolic metabolites, total methylxanthine concentrations (TMC) significantly decreased from the spring drought to the monsoon onset as well as to the monsoon tea harvest (*P*<0.05; Figure 4). Concurrently, total phenolic concentration and antioxidant activity of tea leaves increased from the spring drought to monsoon onset and monsoon tea harvests (Figure 5). No difference was found between the monsoon onset and monsoon for total phenolic concentration but a significant difference was found for antioxidant activity. Overall, seasonal tea harvest period and samples categorized as ‘high quality’ by informants were both found to be significant (*P*<0.02) for secondary metabolites analyzed, total methylxanthine concentration, total phenolic concentration, and antioxidant activity independent of plot effects (soil characteristics and minor elevation differences).

The increase in leaf weight was correlated with a decrease in the concentrations of individual phenolic compounds (CAT, ECG, EGC, EGCG, GA and GCG), TMC, and antioxidant activity (*P*≤0.05). Total phenolic concentration did not significantly change with leaf weight.

### Farmer knowledge, sensory preferences, and tea prices

We found a high degree of agreement between farmer responses to structured survey questions on observed climate patterns and the effects of precipitation variability on tea quality. The eigenvalue ratio between the first and second factors was 10.83∶1 and the average competency (or agreement) score was 0.82±0.13. The first eigenvalue of the informant response matrix accounted for 85% of the total response variance. There was complete consensus for 16 out of 23 questions including agreement that precipitation changes during informants' lifetimes have impacted tea yields, quality, and associated livelihoods.

Coded interview responses found that informants perceive that precipitation patterns have changed during their lifetimes including observations of increased duration (78%), strength (78%), and unpredictability (78%) of rains. Additionally, informants perceive that temperatures have increased (78%) during their lifetimes resulting in fewer cold days (78%), warmer winters (78%), warmer summers (72%), and less frequent occurrence of frost (72%). Informants noted these changes in climate patterns have impacted their tea agro-ecosystems with earlier spring tea harvests (94%) and monsoon tea harvests (78%). Field observations with farmers in December 2012 found the unusual occurrence of a fourth tea harvest with warmer temperatures; tea plants are usually dormant during this period due to colder temperatures.

The majority of informants perceive that precipitation variability impacts tea quality (94%) and yields (81%), although fewer than half agree that temperature variability impacts tea quality (44%) and yields (38%). All informants agreed that quality varies during the three seasonal tea harvests and is inversely related to leaf size. Tea harvested during the dry spring harvest is rated with the highest quality and best sensory preference. Farmers perceive this high quality deteriorates with the arrival of the East Asian Monsoon, when quality and sensory preference is ranked the lowest. Specifically, informants reported that tea harvested during the dry spring harvest has stronger aroma (81%), physiological characteristics (69%), and taste (66%) that many describe as more bitter and bitter-sweet with a sweet lingering aftertaste at the back of the throat referred to as *gaan* (50%). In addition to quality, farmers noted that precipitation levels are directly related to tea growth. Specifically, informants reported that when tea quality is highest during the spring harvest, there is less leaf budding (81%) and tea leaves are drier (88%) and smaller (75%) compared to the monsoon tea harvest season. In addition to direct effects of precipitation variability on tea quality and yields, almost 70% of informants reported that changes in climate patterns have impacted tea quality and yields by altering the quantities and types of pests and weeds in and around tea agro-ecosystems.

**Figure 6:**
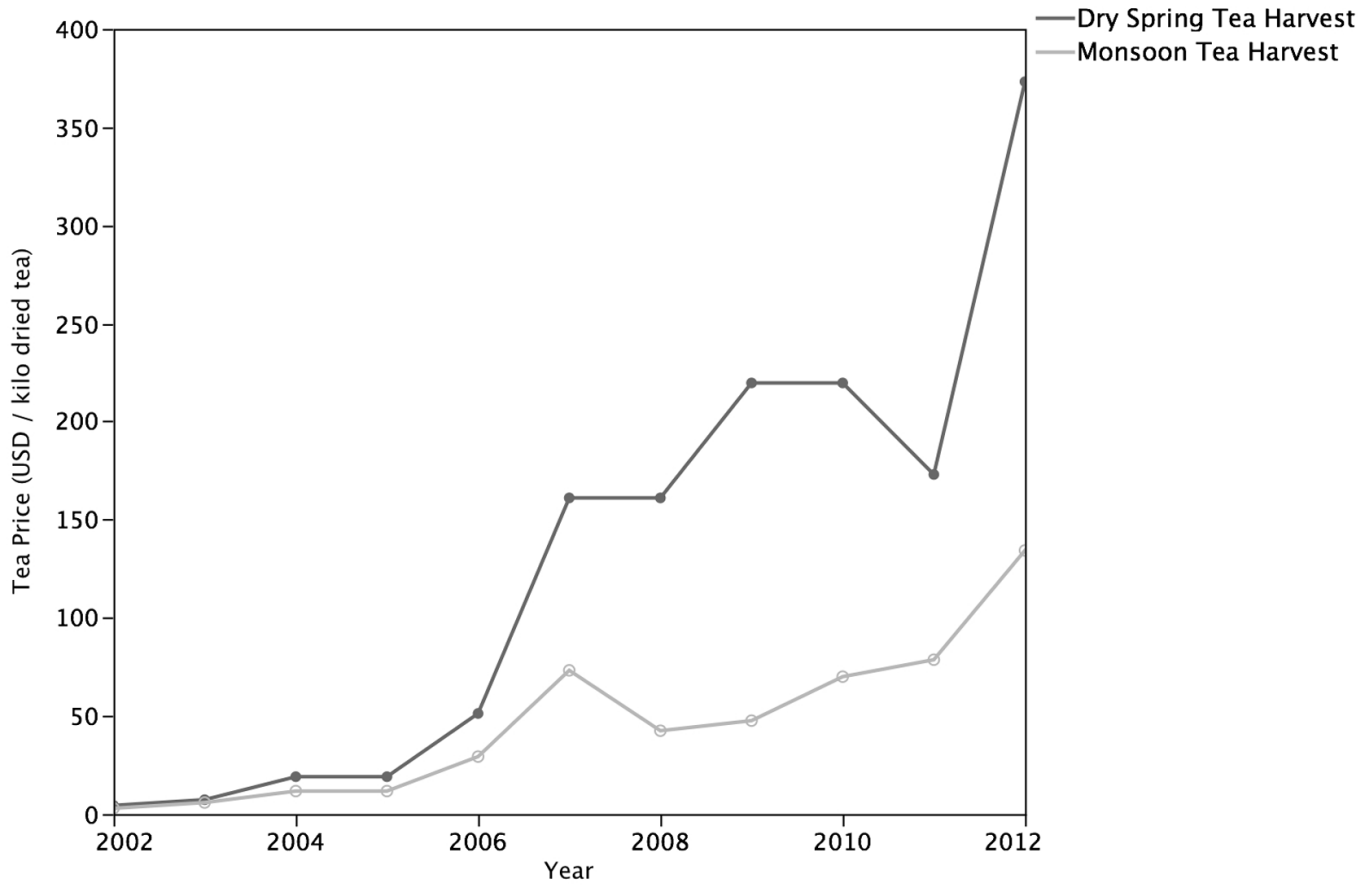
Effects of Precipitation Variability on Tea Prices. Farmers at the study site have experienced an average decrease of 51% in on-farm tea prices received during the Monsoon tea harvests compared to the dry spring tea harvests.

Informants reported that tea traders that buy and sell tea from the study site have the greatest demand for tea harvested during the spring and recognize monsoons as deleterious to tea quality and prices while regarding droughts as positive for tea quality but harmful to yields. Tea samples categorized as ‘high quality’ by informants were significantly correlated (*P*<0.006) with the spring drought tea harvest independent of plot effects. For the past decade, the perceived drop in tea quality and increased leaf growth that occurs with the onset of the East Asian Monsoon has been accompanied with a decrease of 30–50% in livelihoods derived from tea sales. Specifically, for the 2002–2012 period, farmers experienced an average decrease of 51% in on-farm tea prices during the monsoon tea harvests compared to the spring drought tea harvests (Figure 6). Informants (81%) attributed the highest historical tea price that occurred during the dry spring tea harvest of 2012 to an especially high quality crop resulting from the severe drought and not to decreased yields. Only a few farmers (16%) attributed the high spring tea prices to the 25–33% decline in yields resulting from the drought compared to previous dry spring harvests. Regardless of precipitation effects on tea, farmers expressed that climate factors have not negatively impacted their tea sales to date (97%) but have negatively impacted food supply and income from other crops (72%). However, farmers reported concern regarding the drought threshold that is beneficial for tea quality before tea plants loose viability.

All farmers noted having knowledge of management practices to reduce vulnerability of tea crops to climate variability. Management practices employed by farmers at the study site to mitigate climate risk in their agro-ecosystems include cultivating tea from seed rather than from clonal propagules (81%) and growing tea plants as trees rather than as shrubs (50%). Lastly, farmers noted the importance of maintaining canopy coverage (81%) and forested buffer zones around agro-ecosystems (72%) in order to reduce risk in their tea production systems.

### Precipitation variability: historical trends and projected scenarios

**Figure 7:**
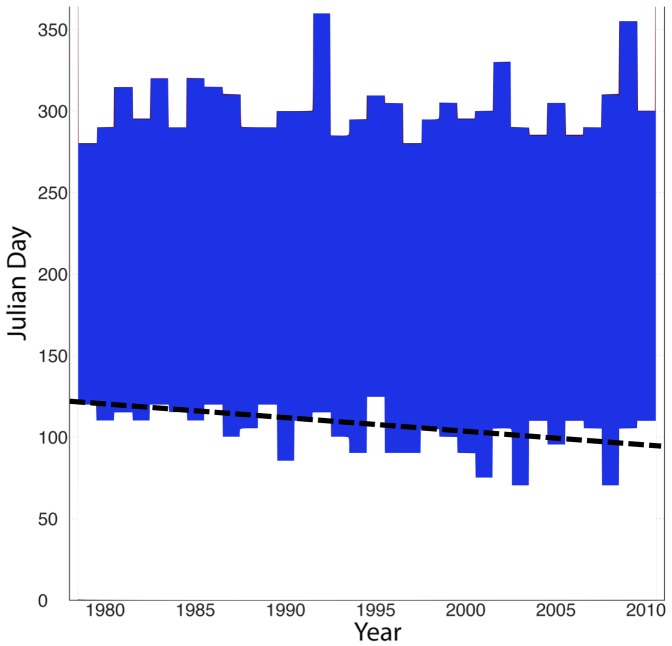
Increased Inter-annual Variability and Total Precipitation. Precipitation data at the study site shows inter-annual variability from 1979 through 2010. The shaded section of the figure represents the period comprising 90% of annual rainfall. In addition to increasing variability since 1990, the monsoon season is arriving earlier as indicated by the downward shift of the trend line.

**Figure 8:**
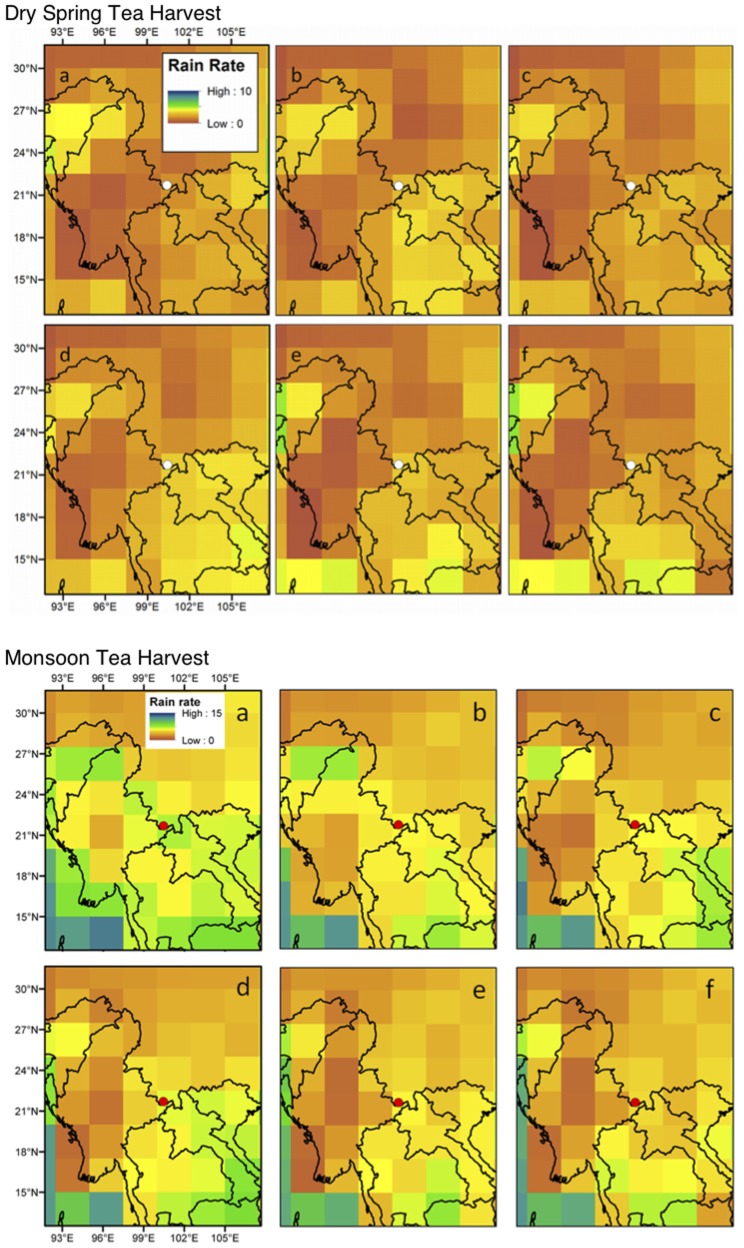
Historical Precipitation Trends During the Dry Spring and Monsoon Tea Harvests. Comparison of average rain rate for dry spring (top) and monsoon (bottom) tea harvests during the following periods: a) 1979–1984, b) 1985–1989, c) 1990–1994, d) 1995–1999, e) 2000–2004, f) 2005–2010. The study site is located at the dot in the middle of each map.

Significant seasonal variation was found in precipitation during the three sampling periods of the spring, monsoon onset, and monsoon tea harvests while no variation was found in temperature. Historical trends of observed precipitation data show inter-annual variability at the study site from 1979 through 2010. The wet season has increased in length since 1990 by starting earlier and/or ending later (Figure 7). Over the past 30 years, mean daily precipitation has increased during the dry spring tea harvest and decreased during the monsoon tea harvest (Figure 8). Climate scenario projections for the 2050–2079 period suggest that mean precipitation for this period is expected to decrease 4–6% during the dry spring tea harvest and increase by 6–8% during the monsoon harvest compared to the historical 1970–1999 period.

## Discussion and Conclusion

This study highlights links between precipitation variability, crop quality, farmer knowledge, sensory preferences, and product prices using tea production and consumption as a model specialty crop system. Compared to the monsoon harvest, the extreme drought sampled during this study was significantly correlated with heightened tea quality on the basis of secondary metabolite concentrations and increased farmer sensory preferences while these quality parameters had an inverse relationship to tea yields. Specifically, increased precipitation from the spring drought to the monsoon rains resulted in a significant increase of up to 50% in tea leaf growth as measured by leaf length and weight. Concurrently, increased precipitation resulted in a decrease of up to 50% in tea functional quality as determined by concentrations of individual catechin secondary metabolites and total methylxanthine concentrations while total phenolic concentrations and antioxidant activity increased. The decrease in measured tea quality parameters during the monsoon matched farmer perceptions and the decrease in tea prices that were up to 50% lower compared to the spring tea harvest. These findings validate farmer knowledge of climate effects on tea quality and rationalize sensory preferences for spring tea as well at its' higher demand and associated higher prices compared to tea from the monsoon harvest. Extrapolating findings from this seasonal study of extreme precipitation variability coupled with historical precipitation trends and projected climate scenarios in order to understand broader climate change suggests that consumers and producers are likely to experience both positive and negative effects as extreme droughts and heavy rains are becoming more frequent with forecasted drier spring seasons and wetter monsoon seasons.

Results of this study concur with previous studies in other geographic areas that altered precipitation has a notable effect on plant performance [18], [28] and significantly impacts both growth and secondary metabolite concentrations of tea plants [29], [30], [31]. The smaller length and weight of tea leaves harvested during the spring drought compared to the monsoon tea harvest in this study supports the widely accepted recognition that lower 3soil moisture content reduces photosynthesis and growth of plants [32], [33]. While soil moisture content is linked to survivability of plants, the spring drought leaves in the present study appeared dry but did not have the physical characteristics of tea leaves under severe water stress suggesting that tea plants can tolerate additional water stress before compromising viability of harvest. Further research is needed to determine precipitation and soil moisture levels that compromise the viability of tea production in various geographic areas and altitudes as well as in agro-ecosystems that follow various modes of management such as tea agro-forests, mixed crop tea gardens, irrigated gardens, and organic production.

This study confirms that rainfall levels are of central importance to tea quality and concurs with previous studies in other climatic zones on the effects of season on tea quality including lower total methylxanthine and catechin concentrations during the monsoon tea harvest when leaf growth is higher compared to the dry spring harvest [31], [34], [35], [36], [37], [38], [39]. For example, Gulati and Ravindranath [31] found that methylxanthine concentrations of tea samples harvested in northeastern India were highest during the early harvest of the dry spring season, lowest during the monsoon season, and improved with the autumn harvest. Further in line with this study's findings, Kottur et al. [38] showed that catechin compounds and overall flavor index of tea harvested in south India were highest during the dry season followed by the pre-monsoon season and were lowest during the monsoon. Similarly, Yao et al. [30] found that catechin compounds were lowest in tea produced during the wet season in Australia while Muthumani et al. [39] reported that the south-west monsoon season resulted in tea of poor quality as determined by biochemical composition including catechins and quality improved with the retreat of the monsoon. Jeyaramraja et al's [37] studies on the impacts of soil moisture stress on tea phytochemicals found that there are optimal drought conditions that upregulate phytochemicals linked to tea quality and water stress beyond this point will result in a decline of these compounds. Further research is needed to determine the optimal soil moisture levels for tea functional quality across various spatial scales and management practices.

While this study did not directly measure volatile aroma compounds, the catechin compounds measured in this study are recognized to be involved in the development of favorable aroma compounds in tea [40], which further explains the higher perceived quality of tea harvested during the dry spring when catechins were the highest. Previous studies support that desirable volatiles and other compounds influencing tea flavor profiles decrease with the monsoon [40], [41]. However, previous work also suggests that unlike the catechins and methylxanthines quantified in this study, some phytochemicals and plant constituents in tea including certain amino acids, crude fiber, moisture content, and chlorophyll increase with wet weather conditions and variably impact the aroma and taste of the final brew [38], [42]. For example, higher moisture content, crude fiber, and chlorophyll of leaves harvested during the monsoon are recognized to decrease the flavor of brewed tea while certain amino acids favorably increase perceived flavor [38]. Further research is needed to assess the effects of climate on tea quality as a function of the approximately 500-1,000 secondary metabolites in tea that both favorably and unfavorably contribute to tea functional quality.

Our results indicate a discrepancy in the trend of changes in individual secondary metabolite concentrations compared to changes in total phenolic concentration and antioxidant activity with increased precipitation of the monsoon tea harvest. Surprisingly, the decrease in individual secondary metabolite concentrations with increased precipitation was not congruent with the trend of increased total phenolic concentration and antioxidant activity. This discrepancy may reflect the fact that larger tissues can produce higher concentrations of phenolic compounds other than the individual phenolic metabolites measured in this study that determine tea functional quality including tannins and other simple phenolics such as glycosylated flavonols, proanthocyanidins, and phenolic acids and their derivatives that also have antioxidant activity [43], [44], [45].

The inverse relationship between tea growth and key functional quality parameters found in this study suggests a possible dilution effect of precipitation. Previous research has identified that enzyme reactions controlling the dynamic metabolic systems in tea leaves are also responsible for changes in tea quality parameters during different seasons, with low enzyme activity in the rainy season [39]. In addition, studies have indicated that the availability of compounds for the formation of soluble solids in tea plants is greater during periods of restricted growth such as the drought [40]. Irradiation effects of higher sunshine during the dry season and lower sunshine hours during the monsoon season could be another reason for the differential accumulation of particular phytochemicals in tea shoots [43]. Increased leaf temperatures during the drought have further been attributed to changes in the synthesis of tea phytochemicals [37]. In addition, processing of tea leaves with greater moisture content harvested in the monsoon season requires greater time during the processing steps of pan-frying, steaming, wilting, and drying and this additional processing collectively contributes to degradation of tea phytochemicals.

The methylxanthines and individual catechins that declined with increased precipitation in this study are key secondary metabolites associated with flavor, health claims, stimulant properties, and other quality characteristics of tea [46]. The catechins are the most abundant secondary metabolites in tea plants comprising approximately 30% of dried leaf extract and are also the most biologically active constituents of tea [13]. The consumption of catechins in humans is inversely associated with the incidence of chronic disease including cardiovascular diseases, bone and skin health, inflammation, oxidative stress, cancer, neurodegenerative condition, and the metabolic syndrome [47]. The significant decrease in catechin concentrations during the monsoon suggests decreased consumption of these dietary phytonutrients from tea harvested during forecasted periods of increased precipitation and increased consumption of these dietary phytonutrients during periods of decreased precipitation and subsequent implications for human health.

At the market level, consumer perceptions of the decreased functional quality of tea via its' sensory characteristics during the monsoon harvest [7] drive the decline in tea prices [38]. Tea harvested during the dry season is recognized to fetch higher prices than tea harvested during the monsoon season in major tea production countries [38]. Previous reports of Chinese tea production have documented that high quality tea is primarily produced during the dry season and accounts for approximately 80% of total production value while accounting for only 40% of total production quantity [48]. Projected climate scenarios of increased extreme climate events are thus expected to impact the production value and prices of tea and directly impact farmer livelihoods. These dynamics are important to understand towards designing appropriate climate adaptation strategies and policies that best benefit both producers and consumers in the context of global environmental change.

The large degree of agreement found in the cultural consensus analyses regarding farmer knowledge of climate variability and effects on tea highlights the agro-ecological knowledge [49], [50], [51] of the study-site community and the customary natural resource institutions and social networks that maintain this knowledge. The maintenance of ecological knowledge in regards to global environmental change is a recognized factor to enable resilience and adaptation to environmental variability [52]. Ultimately, the adaptive capacity of a socio-ecological system determines the extent of climate effects on natural resources, livelihoods, and wellbeing. This study supports that smallholder farmers at the study site have knowledge that climate drives a decline in tea quality either through personal sensory experiences and/or interactions with the market. Farmers at the study site further have knowledge through their agreement that tea cultivated from seed and maintained as trees are more resilient to the effects of precipitation variability compared to tea plants cultivated from clonal propagules and maintained as shrubs. In addition, farmers agree that tea plants grown in diverse, multi-storied agro-forests with canopy coverage and forest buffers are more resilient to the effects of precipitation variability compared to tea plants grown in monoculture terraced plantations. Previous studies have validated that clonal tea shrubs are particularly susceptible to drought effects and show severe water stress during the dry season compared to tea cultivated from seed [53]. Despite such findings, agricultural extension programs and enterprises throughout tea-growing areas of China and worldwide continue to promote the cultivation of tea in monoculture plantations using clonal propagules. Further research is needed to examine how variable management modes of production and practices such as agroforestry systems may buffer the negative impacts of climate variability. For example, previous studies have identified agro-forestry as an adaptive strategy against potential microclimate extremes in several specialty crop systems such as coffee production [54], [55]. Studies are needed to identify the variables that promote farmer knowledge of climate effects on crop quality and associated management adaptation. Policies and plans may than use this evidence to foster the variables promote farmer knowledge and adaptive management towards increased resilience of tea agro-ecosystems to global environmental change.

Findings from this study are in agreement with previous research showing vulnerability of tea-producing areas to changing precipitation patterns with consequences for environmental, economic, and social factors [56], . For example, tea production in Sri Lanka is forecasted to experience more intense dry and wet periods with anticipated implications for top-soil erosion, increased agro-chemical run off, and irreparable yield loss that will subsequently have negative socio-economic repercussions for the laborers that compromise the country's main net foreign exchange earner [56]. However, climate deviations in other tea-producing areas such as a rise in mean temperature during the cold season in Northern India have been reported as beneficial for an early tea crop and extended harvest [57]. Taken together, the totality of evidence emphasizes differential responses of tea production systems across geographic scales.

Extrapolating findings from this seasonal study, previous precipitation trends, and projected climate scenarios to understand broader climate change suggests that farmers are facing increased intra-annual livelihood variability with the increased prevalence and intensity of extreme droughts and heavy rains. The increased length of the monsoon season since 1979 as well as increased precipitation during the dry seasons indicates that tea quality and prices at the study site have been adversely impacted. Looking forward at climate scenarios for the 2050–2079 period during when drier spring harvests and wetter monsoon harvests are projected implies both beneficial and adverse impacts to tea quality within a single year. Changes in the concentrations of phytochemicals have the potential to alter sensory experiences, physiological effects, and consumer willingness to pay for tea products. Such shifts are thereby likely to impact tea prices and income received by tea famers in China and the approximately 60 other tea-producing countries worldwide.

The effects of extreme climate events on tea crops are likely to intensify as prolonged seasonal effects over time may exceed thresholds for plant survival. Further, total effects on crops may intensify with additional shifting dynamics within agro-ecosystems including pest pressures and weeds that farmers reported to already experience in this study. For example, temperature and rainfall changes can shift pest geographic ranges and result in new immigration of pests into tea production areas [48]. Alterations of wind, humidity, and temperatures would present additional dynamics. The feedbacks between these abiotic and biotic changes may alter the geographic range of tea plants as well as those classified as high quality. At the same time, it is important to consider and understand how tea crops may evolve with these shifts in climate variability and accompanying abiotic and biotic changes in tea agro-ecosystems.

In conclusion, this study provides evidence for the relevance of examining crop quality as well as yields in research on the potential effects of climate change on production to facilitate climate adaptation for sustainable crop systems. This study validates farmer perceptions of extreme drought effects on tea quality and rationalizes the higher demand and prices for the spring tea harvest compared to the monsoon tea harvest. Farmer perceptions of the multiple climate variables that impact tea agro-ecosystems points to the need to look beyond only the early onset of the spring season and plant budding that dominates the literature on plant responses to climate change. Lastly, further research is warranted in tea-producing areas located in diverse geographic and climatic zones to compare precipitation effects on tea quality over various spatio-temporal scales and management practices to identify variables that foster resilience of tea production and sustain farmer livelihoods and consumer wellbeing.
